# An alginate-based encapsulation system for delivery of therapeutic cells to the CNS

**DOI:** 10.1039/d1ra08563h

**Published:** 2022-02-01

**Authors:** Despoina Eleftheriadou, Rachael E. Evans, Emily Atkinson, Ahmed Abdalla, Francesca K. H. Gavins, Ashleigh S. Boyd, Gareth R. Williams, Jonathan C. Knowles, Victoria H. Roberton, James B. Phillips

**Affiliations:** UCL Centre for Nerve Engineering, University College London London UK; UCL School of Pharmacy, University College London London WC1N 1AX UK jb.phillips@ucl.ac.uk; Biomaterials & Tissue Engineering, UCL Eastman Dental Institute London UK; UCL Institute of Immunity and Transplantation, Royal Free Hospital London UK

## Abstract

Treatment options for neurodegenerative conditions such as Parkinson's disease have included the delivery of cells which release dopamine or neurotrophic factors to the brain. Here, we report the development of a novel approach for protecting cells after implantation into the central nervous system (CNS), by developing dual-layer alginate beads that encapsulate therapeutic cells and release an immunomodulatory compound in a sustained manner. An optimal alginate formulation was selected with a view to providing a sustained physical barrier between engrafted cells and host tissue, enabling exchange of small molecules while blocking components of the host immune response. In addition, a potent immunosuppressant, FK506, was incorporated into the outer layer of alginate beads using electrosprayed poly-ε-caprolactone core–shell nanoparticles with prolonged release profiles. The stiffness, porosity, stability and ability of the alginate beads to support and protect encapsulated SH-SY5Y cells was demonstrated, and the release profile of FK506 and its effect on T-cell proliferation *in vitro* was characterized. Collectively, our results indicate this multi-layer encapsulation technology has the potential to be suitable for use in CNS cell delivery, to protect implanted cells from host immune responses whilst providing permeability to nutrients and released therapeutic molecules.

## Abstract 2

Novel composite cell encapsulation system: dual-layer, micro-scale beads maintain cell survival while releasing immunomodulatory FK506 in a sustained manner. This biotechnology platform could be applicable for treatment of CNS and other disorders.

## Graphical abstract

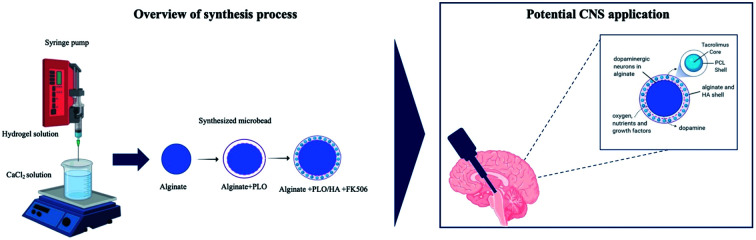

## 1. Introduction

As life expectancy has increased dramatically over the past century, conditions associated with ageing have become more prevalent.^1^ Neurodegenerative diseases, such as Alzheimer's disease, and Parkinson's disease (PD), represent a daunting worldwide challenge for society and healthcare providers. These disorders are associated with extensive loss of neuronal cells, reflecting cellular demise, and are clinically characterized by progressive cognitive, motor, and behavioural impairments. Final stage patients are left bed-ridden and dependent on specialist care.^2–4^

Recent advances in the area of regenerative medicine have yielded new opportunities to develop targeted therapies. Two of those, cell- and neurotrophic factor delivery, have been trialed in humans to-date. The first aims at replacing lost neurons and functional reinnervation.^5^ In the case of PD, this approach involves transplanting new cells capable of forming network connections and producing dopamine.^6,7^ However, both pre-clinical and clinical evidence shows high rates of cell death after implantation, which could be partially attributed to the hostile mechanical and chemical host tissue environment that cells encounter and activation of the host immune system.^8–10^ For instance, only 1% to 20% of grafted neurons survive in animal models of PD.^11–13^

Deficiency of neurotrophic factors, such as cerebral dopamine neurotrophic factor (CDNF), neurturin and glial cell line-derived neurotrophic factor (GDNF) has also been related to neurodegenerative diseases. Therefore, delivery of these factors has been the basis of the second neuro-regenerative strategy which, instead of replacing lost neurons, focuses on employing neurotrophins to enhance the growth and function of viable neurons in the affected areas.^14,15^ Despite early optimism, this approach has also proved challenging, mainly due to the short half-life of proteins *in vivo* and challenges with targeting delivery, with clinical outcomes not meeting the expectations of the preclinical data.^16,17^

An alternative strategy for targeted dopamine replacement and neuroprotection could be achieved by the delivery of therapeutic cells which can release dopamine and secrete neurotrophic factors without needing to integrate synaptically and form cell–cell connections with host cells.^18,19^ Using this approach would enable therapeutic cells to remain permanently encapsulated following transplantation, separating them physically from direct interaction with host cells whilst enabling the exchange of soluble factors required to sustain the engrafted cells and elicit their therapeutic effects. Established protocols exist for the reliable generation of neural progenitors which can either release dopamine or be used as delivery vehicles for trophic factors such as GDNF following stable transfection.^20,21^ A commonly applied technology of immunoisolation is microencapsulation of cells in spherical beads made of hydrogels that allow passage of oxygen and nutrients while protecting transplanted cells. This inhibits immune recognition by restricting cellular interactions and leads to a significant increase in the survival of transplanted cells.^22^ While encapsulated cell therapy has been extensively studied for the treatment of conditions like diabetes,^23,24^ the use of this approach in therapy for CNS disorders remains limited.^25^ Alginate based 3D platforms have been shown to not interfere with the dopaminergic potential of encapsulated cells. Encapsulation in alginate beads facilitated the early onset of neuronal dopamine generation compared with conventional 2D systems, with 3D differentiated cells showing higher dopamine secretion.^26^ A recent clinical trial that studied the efficacy of immunoprotected (alginate-encapsulated) porcine choroid plexus cells for xenotransplantation in patients with PD showed that this technology can be relatively safe and well-tolerated.^27^

Still, an outstanding issue with microencapsulation that requires resolution is the inability of this passive protective barrier to efficiently protect cells from exposure to cytokines and other small diffusible cytotoxic molecules produced by stimulated immune cells.^28^ Thus, surface modification,^29,30^ or immobilization and release of immunomodulatory molecules might also be considered an effective strategy to suppress the host immune response upon implantation.

Here, we have developed a composite cell encapsulation system, consisting of dual-layer micro-scale beads that can maintain cell survival while concurrently being able to release an immunomodulatory compound in a sustained manner. The use of multi-layer biotechnology allows the design of cell encapsulation vehicles with optimal degradation rates and desirable physicochemical properties. The proposed system consists of (a) an inherently non-degradable core for cell encapsulation and (b) an outer hybrid polymer layer that will ultimately degrade for FK506 encapsulation and controlled delivery. This approach can allow the transplantation of cells that release therapeutic soluble factors while protected from the host immune response.

For the core of the beads, we chose alginate; an inert, readily available, nontoxic biomaterial with tunable properties. Alginate is one of the most frequently used biomaterials for cell encapsulation,^22^ mainly due to its ability to form gels under conditions suitable for cell survival.^31^ For the outer layer, a composite of alginate and hyaluronic acid (HA) was selected. Both alginate and HA can be manipulated to present mechanical properties that comply with those of native brain tissue.^32^ Alginate has been shown to not interfere with the survival, differentiation, maturation or growth factor secretion of encapsulated neuronal cells.^26,33,34^ Moreover, it has a low capacity to support cell–matrix interaction owing to the lack of suitable mammalian cell adhesion molecules, and low protein adsorption capacity.^26,35,36^ HA is a biocompatible and bioresorbable material that plays a widespread role in cellular signaling, differentiation, proliferation, and cell migration in the CNS.^37,38^ Survival of encapsulated cells in alginate beads was demonstrated here using human neuroblastoma SH-SY5Y cells, a catecholaminergic neuronal cell line commonly used *in vitro* in models of neurodegenerative disease.^39^

To further reduce the host tissue immune response following transplantation, we incorporated FK506, a widely used potent immunosuppressant, into the outer layer of the encapsulation system. FK506 is a macrolide drug that exerts its immunosuppressive effects by binding to FK506-binding proteins and ultimately preventing T-cell activation.^40,41^ Although FK506 is commonly used in allograft transplant rejection prophylaxis,^42^ systemic administration has been associated with nephrotoxicity, neurotoxicity, and gastric disturbances.^43^ These side-effects could potentially be reduced using a local/implantable delivery system. Compared to traditional dosing regimens, implantable formulations require a considerably lower pharmaceutical dosage and allow the local drug concentration to remain within the therapeutic window over an extended period, thus minimizing side effects and ensuring efficacy. Furthermore, the use of hydrogels can help to achieve continuous drug release at isolated sites, where it is difficult to maintain nanoparticles for long time periods.^44,45^ Therefore, FK506 was incorporated into the outer layer of alginate beads either in soluble form or in nanoparticles. To provide appropriate stability and release kinetics, the nanoparticles were formed using a core–shell approach through coaxial electrospraying of poly(ε-caprolactone) (PCL) with FK506. This demonstrates a novel method for protecting implanted cells through localized FK506 release as part of a cellular encapsulation system suitable for delivery of therapeutic cells to the CNS.

## 2. Materials and methods

### 2.1. Reagents

Unless otherwise stated all cell culture materials and chemical reagents were purchased from Sigma Aldrich (Gillingham, UK) or Thermo Fisher Scientific (Loughborough, UK). Alginic acid sodium salt powder was purchased from Sigma Aldrich (Gillingham, UK). FK506 was obtained from Abcam (Cambridge, MA, USA). MagCellect Rat CD4 + T cell isolation kits were sourced from R&D systems (Minneapolis, MN, USA). Cell activation cocktail was provided by Biolegend (San Diego, CA, USA). A FK506 enzyme linked immunosorbent assay (ELISA) kit was purchased from Abnova (Taipei City, Taiwan). Running buffer and calibration beads for flow cytometry were obtained from Miltenyi Biotec (Gladbach, Germany).

Collection and use of tissue from animals were conducted in accordance with the UK Animals (Scientific Procedures) Act (1986) and the European Communities Council Directives (86/609/EEC) and approved by the UCL Animal Welfare and Ethical Review Body.

### 2.2. Material preparation and characterisation

#### 2.2.1. Preparation of alginate beads

**Fig. 1:**
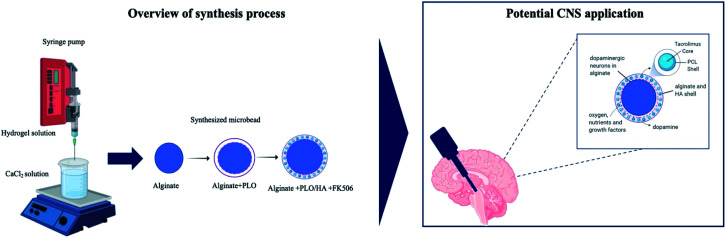
Schematic illustration of the process used to synthesize the multilayer alginate beads.

Sodium alginate was dissolved in Dulbecco's Modified Eagle's medium (DMEM), under sterile conditions, to form final concentrations of 1.5% or 2% (w/v). The mixture was extruded into a 102 mM calcium chloride solution from a syringe equipped with a needle (15–27 gauge), either drop-wise or using a syringe pump at a flow rate of 25 ml h^−1^. Formed beads were left in a CaCl_2_ bath for 25 min for ionic crosslinking to occur. For cell encapsulation, cells at the desired density (2.5 × 10^5^ to 2.5 × 10^6^ cells per ml) were dispersed in the alginate solution. Following the gelation step, the CaCl_2_ solution was removed and the beads were washed twice with 0.9% (w/v) saline. Saline was then replaced with cell culture medium or coating solution. For the latter, beads were suspended in 0.1% w/v poly-l-ornithine (PLO) solution for 10 minutes, washed with saline and then 0.3% w/v alginate (alginate + PLO beads) or 0.3% w/v alginate-0.3% w/v HA solution (alginate + PLO/HA beads) was added to neutralize the residual positive charge. After that, beads were resuspended in the CaCl_2_ solution and allowed to crosslink for an additional 5 minutes. Then they were washed with saline to remove the excess Ca^2+^ and suspended in culture media (Fig. 1). The diameters of the beads under different conditions were imaged (Nikon 200) and measured using Image J (National Institutes of Health, Bethesda, MD, USA).^46^ For each group, 10 randomly selected beads were measured.

For imaging purposes, DAPI (4′,6-diamidino-2-phenylindole) and FITC positive PCL nanoparticles were incorporated in the core and shell of alginate + PLO/HA beads respectively. Multichannel image acquisition was performed using an inverted fluorescence microscope (EVOS Fl) with 4× objective. Confocal microscopy (LSM710, Zeiss, Oberkochen, Germany) was used to capture z-scans of a bead section using a 40× lens.

#### 2.2.2. Swelling degree of alginate beads

The swelling degree of the beads was determined as previously described.^47^ Four beads per sample were immersed in physiological saline and culture media, until the maximum swelling equilibrium was attained.

Absorption properties were evaluated by estimating the weight swelling ratio (SW) based on eqn (1),

1 SW = (*W*_swollen_ − *W*_dry_)/*W*_dry_,

where, *W*_swollen_ is the weight of the swollen sample and *W*_dry_ is the weight of the dry sample.

#### 2.2.3. Diffusion studies of alginate beads

In order to assess the diffusion properties of dissolved molecules within alginate beads, FITC labelled dextran (MW 44 or 150 kDa) was added to the final alginate solution (5 mg ml^−1^ of alginate) before gelation. After formation, beads were washed twice with 2 ml of Dulbecco's phosphate-buffered saline (DPBS), and the solution was collected in order to measure the entrapment efficiency. The beads were immersed in fresh DPBS, and aliquots of 2 ml were collected to measure the fluorescence of released dextran. A calibration curve for each MW dextran *λ*_ex_ = 490 nm, *λ*_em_ = 535 nm was made. At 2, 4, 24, 48, 96 and 168 h, aliquots were collected, and the amount of FITC-dextran released was determined by measuring the fluorescence intensity of the supernatant at a given time *versus* the amount of the dextran loaded in the beads at time 0.

#### 2.2.4. Rheology

Rheological properties of alginate hydrogel samples were measured on a CVO Rheometry System (ACVO, Bohlin Instruments, UK) using 40 mm diameter plate geometry. The rheology test was performed at 37.5 °C in a constant-temperature environment maintained using an open-bath circulator with stainless steel bath (DC-10, Thermo Haake®, UK). Gels were prepared within the rheometer, and a mass ratio of 1 : 1 crosslinker to polymer solution was maintained constant for all measurements. To determine the linear viscoelastic region of hydrogels, separate strain sweep tests were made. Based on these results, a common strain value was chosen and later used to record viscoelastic properties during oscillatory experiments at a fixed strain of 0.01%, which was within the linear region, under constant frequency of 1 Hz.

#### 2.2.5. Dynamic mechanical analysis (DMA)

Compressive DMA analysis of hydrogels was performed at 21 °C using an ElectroForce 3200 instrument (ElectroForce 3200, TA Instruments, New Castle, DE, USA). 100 μl alginate hydrogels of different alginate compositions were prepared using 24-well transwell inserts (ThinCerts™, 1 μm diameter pore size, Greiner Bio-One, Kremsmünster, Austria), equilibrated at 21 °C for 30 min then mean thickness was calculated using an optical angle meter (Cam 200, KSV Instruments, Helsinki, Finland). Hydrogels then underwent a measured DMA cycle based on an established protocol.^32^ This consisted of an ascending 1–70 Hz frequency sweep with 2% dynamic mechanical amplitude. Once the sweep had been completed, a 1 Hz validation frequency was then repeated to assess for signs of mechanical destruction. Likewise, thickness measurements were repeated to check for evidence of geometric change.

#### 2.2.6. Preparation of FK506 loaded nanoparticles

PCL particles with a defined core–shell morphology were produced by coaxial electrospraying. For the shell, 45 kDa PCL was dissolved in mixture of 2,2,2-triflouroethanol (TFE) and deionized water (DI) 9 : 1 v/v to obtain a 5% w/v solution. An FK506 core solution was dissolved in ethanol to a final concentration of 0.1% w/v. Both solutions were loaded into disposable plastic syringes, mounted to feed a stainless-steel coaxial spinneret (inner/outer needle internal diameters: 0.5/1.0 mm). The solutions were ejected through the spinneret using two separate syringe pumps (KDS100, Cole-Parmer, London, UK) at constant ratio flow rates of 1 : 10 core to shell (core; 0.01 ml h^−1^, shell; 0.1 ml h^−1^). The spinneret was connected to the positive electrode of a high-voltage DC power supply (18–20 kV, HCP35-35000, FuG Elektronik, Schechen, Germany), and nanoparticles were collected on a grounded stainless-steel plate 18 cm from the tip. To improve the recoverability of the particles, the collector plate was pre-coated with a film of 5% w/v polyvinylpyrrolidone (PVP) dissolved in ethanol. The film was allowed to dry in air before spinning commenced. For fluorescent NPs, instead of FK506 the core consisted of fluorescein sodium salt dissolved in ethanol (5 mg ml^−1^).

#### 2.2.7. *In vitro* release studies

Drug release from FK506-loaded PCL nanoparticles (NPs) was performed by suspending 0.1 mg of NP powder in 10 ml of DPBS at 37 ± 1 °C. Aliquots of 2 mL were withdrawn with a syringe and replenished with pre-heated DPBS. Insoluble solid NPs were removed by centrifugation (13 000 rpm, 1 min), and the remaining solution was analysed using UV-Visible spectroscopy (Lambda 25, PerkinElmer, Beaconsfield, UK) at 205 nm. Standard purified FK506 concentrations (0–200 ng ml^−1^ in DPBS buffer pH 7.4) were used to generate a calibration curve. For NPs embedded in the outer layer of ALG + PLO/HA beads, sampling was performed as previously described, while FK506 release was quantified using an ELISA kit according to the manufacturer's protocol. The encapsulation efficiency of the PCL nanoparticles was assessed following the *in vitro* release study. The nanoparticles were isolated by centrifugation as mentioned earlier and burst with pure acetonitrile. A calibration curve was plotted using acetonitrile and the remaining tacrolimus was determined using the UV spectrometer. Then, the encapsulation efficiency of the particles was determined using the following equationEncapsulation efficiency (%) = (encapsulated drug (mg)/amount of drug in feedstock (mg)) × 100%.

#### 2.2.8. Electron microscopy

The morphology and structural features of PCL nanoparticles were examined by scanning electron microscopy (SEM) (FEI Quanta 200 FEG SEM, Thermo Fisher Scientific, Waltham, MA, USA). SEM specimens were prepared by dispersing the NP powder onto aluminium stubs (TAAB Laboratories, Berks, UK) with carbon-coated adhesive tabs, followed by sputter-coating with 10 nm gold for 2 min (Q150R coater, Quorum, Laughton, UK) to enhance conductivity.

For transmission electron microscopy (TEM), samples of liquid NP suspensions were dropped with a Pasteur pipette onto a carbon/formvar coated copper grid. After 15 s excess sample was blotted off with filter paper. Then a drop of stain (1% uranyl acetate) was added if required and blotted after 15 seconds. The grid was placed into a specimen holder and inserted into a Philips/FEI CM 120 BioTwin TEM (FEI Company, Hillsboro, OR, USA) for imaging at 120 kV.

### 2.3. Cell culture

#### 2.3.1. SH-SY5Y culture

SH-SY5Y neuroblastoma cells (Sigma Aldrich, Gillingham, UK; cat no. #94030304) were maintained in 1 : 1 v/v Hams F12 : Eagle's Minimum Essential medium (EMEM) media supplemented with 1% non-essential amino acid solution, 2 mM l-glutamine, 15% v/v fetal bovine serum (FBS) and 1% v/v penicillin/streptomycin (P/S). Cells were passaged when 70–80% confluency was reached by trypsinization, centrifugation at 100 × *g* for 5 minutes and re-suspension in fresh media, then seeded at the desired density. Flasks were kept in a humidified incubator at 37 °C with 5% CO_2_ in air. To test viability, cells were encapsulated in 2% alginate beads produced using a 21 G needle and syringe pump for reproducibility. Cellular beads were then transferred to a 6-well plate and cultured for further analysis.

#### 2.3.2. Live/dead assay

To assess cell viability, cultures were stained using the Syto 21/propidium iodide (PI) double cell staining kit which allows for the simultaneous staining of viable and dead cells. Treatment medium was removed from the 6-well plates, which were then washed three times with 1.0 ml of media (37 °C). Subsequently, 500 μL of Syto 21/PI solution (1 : 1000 v/v dilution) was added and plates were incubated for 15 min at 37 °C then washed briefly with 1.0 ml of culture media. Finally, an additional 1.0 ml of culture media was added to each well prior to image acquisition. For cell viability, multichannel image acquisition was initially captured using rhodamine (for propidium iodide – PI) and fluorescein isothiocyanate (FITC; for Syto21) filters in a fluorescence microscope (Zeiss-Axio Lab. A1, Zeiss, Oberkochen, Germany) with 20× objectives. Manual cell counting of FITC-stained cells in five pre-selected areas based on a sampling protocol led to the determination of the total number of cells and calculation of the percentage of cell viability.

#### 2.3.3. Primary rat T-cell isolation, purification, and culture

T-cells were isolated from the spleens and lymph nodes of 8 week male Sprague Dawley rats. Briefly, the isolated tissue was teased apart in order to generate a single cell suspension which was then passed through a 70 μm strainer to remove any cell clumps and/or debris. Cellular suspension was exposed to a NH_4_Cl lysis buffer (RBC buffer) for the preferential lysis of red blood cells, yielding intact T-cells. Isolation of CD4 + T-cells was performed using a MagCellect Rat CD4 + T Cell isolation kit then T-cells were cultured in complete RPMI-10 medium (1% v/v P/S, 1% v/v l-glutamine, 1% v/v essential amino acids, 10% v/v FBS) for up to 6 days in a humidified incubator at 37 °C with 5% CO_2_.

#### 2.3.4. T-cell proliferation assay and flow cytometry

Purified rat CD4 + T-cells were stained with 5 μM intracellular fluorescent dye (CellTrace™ CFSE) for 20 min and then plated at a density of 10^6^ cells per well in a 24-well plate. Cells were then stimulated by 2 μL of PMA (phorbol 12-myristate-13-acetate), ionomycin, and protein transport inhibitor (Brefeldin A) (cell activation cocktail) for 6 hours. Activated cells were then harvested, centrifuged to remove the activation cocktail, and exposed to different biomaterial-based stimuli. T-cell proliferation was determined 4 days later by flow cytometry analysis of CFSE fluorescence intensity. Cells were washed with 1 ml phosphate-buffered saline (PBS) and centrifuged at 400 × *g* for 5 minutes, the cell pellet was resuspended in PBS then analysed using a MACSQuant Analyzer 10 flow cytometer (Miltenyi Biotec Inc. Auburn, CA, USA). MACSQuant running buffer was used for the analysis and MACSQuant calibration beads were used to calibrate the equipment. Quantitative analysis of the T-cell response was performed by FlowJo software (FlowJo, Ashland, OR, USA) using an in-built proliferation modelling tool.

#### 2.3.5. Data analysis

GraphPad Prism was used for data analysis. Normality was determined using Shapiro–Wilk tests and if there was not a normal distribution then a non-parametric Mann–Whitney test was used. If a normal distribution was shown then either a one-way statistical analysis of variance (ANOVA) or a two-way ANOVA followed by a Dunnett's, Tukey's or Bonferroni multiple comparison test, was performed. Statistical significance was defined as *p < 0.05, **p < 0.01, ***p < 0.001 and ****p < 0.0001.

## 3. Results and discussion

### 3.1. Developing materials for encapsulating therapeutic cells

**Fig. 2:**
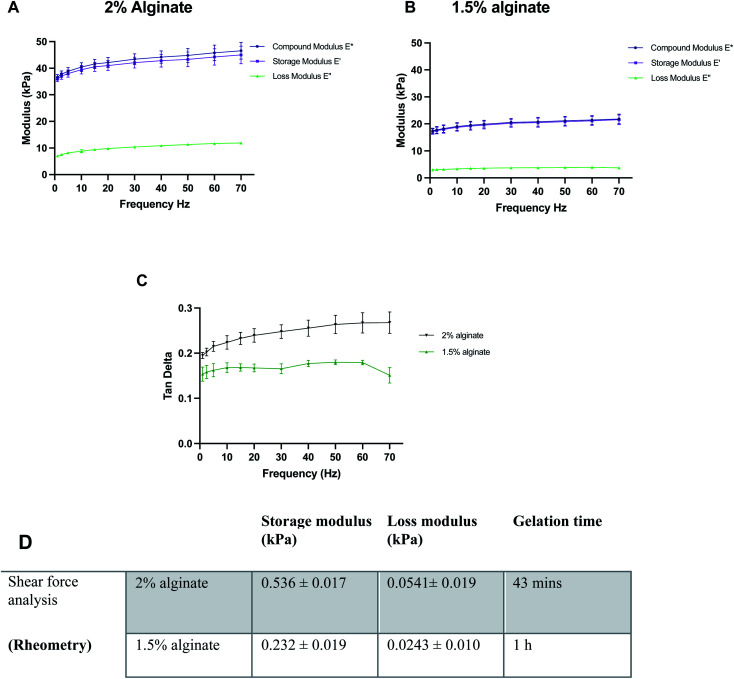
Characterisation of the mechanical properties of different alginate formulations: compressive dynamic mechanical properties of different concentrations of alginate; (A) 2% alginate, (B) 1.5% alginate and (C) tan delta for both conditions. (D) Overview of rheological properties of alginate specimens. Data expressed as means ± SD, *N* = 3.

Before the synthesis of the beads, mechanical properties of alginate were optimised using 100 μl alginate gels cast in ThinCerts™ in 24-well plates to allow for easy testing. DMA analysis revealed that alginate formulations can be benchmarked and tuned to mimic the stiffness properties of the brain, by adjusting the concentration of polymer. Moreover, the results verify the viscoelastic behaviour of the hydrogels as can be seen by the high storage modulus and low loss modulus (Fig. 2A–C).

DMA was performed at 23 °C. However, under physiological conditions, the alginate gels could be mildly stiffer than measured; a 10% increase in the stiffness of alginate gels from 23 °C to 37 °C has been previously reported.^48^ Thus, the temperature for rheological measurements was set to 37.5 °C. For rheological assessment, single frequency oscillatory tests were conducted to evaluate the time-dependent viscoelastic shear behaviour of selected alginate hydrogels. The variation in viscosity was determined as a function of time. Hydrogels were tested at a constant oscillation frequency of 1 Hz, which is within their linear viscoelastic region. The end of the crosslinking process was defined as the time point where six consecutive measurements did not differ by more than 0.5%. Both 1.5% w/v and 2% w/v alginate hydrogels exhibited a much higher storage modulus than their respective loss modulus, indicating that the materials behave like a viscoelastic gel. Moreover, in all formulations, the concentration of crosslinker was found to be sufficient and led to the formation of fully gelled alginate gels. Higher alginate concentration resulted in shorter gelation time. Gelation was completed at 43 min and 1 h for 2 and 1.5% w/v alginate hydrogels respectively (Fig. 2D).

**Fig. 3:**
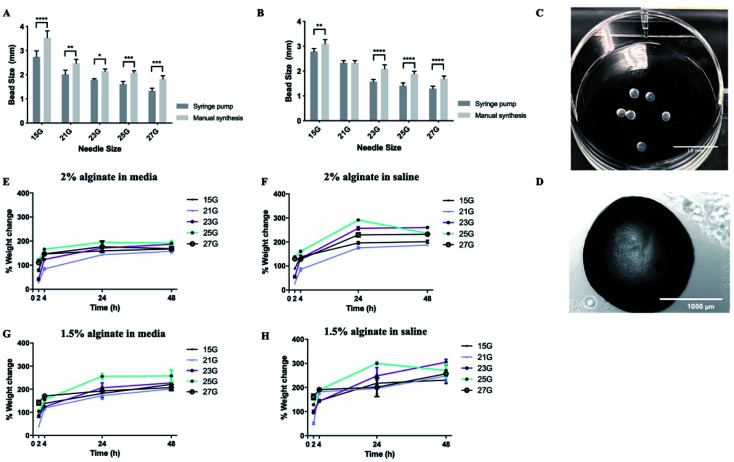
Alginate beads display favorable swelling properties. Alginate beads made using different gauge needles with ejection driven either by syringe pump or manual pressure. (A) Effect of synthesis method for 2% alginate beads. (B) Effect of synthesis method for 1.5% alginate beads. (C and D) Representative images of alginate beads. (E) Swelling ratio of 2% beads in media. (F) Swelling ratio of 2% beads in saline. (G) Swelling ratio of 1.5% beads in media. (H) Swelling ratio of 1.5% beads in saline. Data expressed as means ± SD, *N* = 3 (4 beads per repeat). Analysis was performed *via* two-way ANOVA followed by Bonferroni's multiple comparison test, where *p < 0.05, **p < 0.01, ***p < 0.001 and ****p < 0.0001.

Subsequently, synthesis methods were optimized to control the morphological properties of the alginate beads which is another major influencer of the functional survival of encapsulated cells.^49,50^ Beads displayed varying sizes and swelling rates, depending on the synthesis methods and experimental conditions. Of the methods tested, manual synthesis was the least technically challenging.^51^ However, compared to dropwise dispersion from the syringe by hand, beads generated using a syringe pump exhibited less variability in their size. Additionally, in terms of translational potential, the robustness of the automated bead-generation system allows for further scaling up of the process. While the size of the beads makes their delivery with narrow cannulae problematic, it could be preferable for CNS therapy where large-sized retrievable microbeads may be more suitable for safety reasons in case of complications.^52,53^ Beads synthesized from 2% w/v alginate solution were characterized as having smaller diameters than those from 1.5% w/v when all the other parameters of the synthesis protocol remained the same (Fig. 3A and B). It should be noted that, in all experimental conditions, alginate beads displayed a rather smooth surface without visible defects, as identified by light microscopy (Fig. 3C and D). This is important as broken beads or beads with a rough morphology have been previously linked to the protrusion of cells^35^ and inflammatory responses.^54^

With regards to the swelling ratio, this was significantly higher for beads immersed in saline solution than those in media, with the effect being more pronounced in the case of beads composed of 1.5% w/v alginate (Fig. 3E–H). The swelling behaviour of the beads was also influenced by the gauge of the needle used to produce them, with small-diameter beads exhibiting higher swelling rates, due to their greater surface area. Previous research suggests hydrogels that promote neural survival are more likely to exhibit swelling (up to 366%), which could be a reflection on the extracellular ion environment that is favorable to neurons.^55,56^ Therefore, some degree of swelling is acceptable. Another important consideration is the effect of pH on the stability of the alginate beads. Local acidosis is observed in several neurological conditions and is often related to neuroinflammation. However, the potential reduction of pH is unlikely to cause the degradation of alginate microcapsules. Previous research demonstrated that alginate beads remain intact between pH = 1 to pH = 7 but exhibit some swelling at pH > 3 due to loss of negative charges (p*K*_a_ ≈ 3.5).^57^

Upon optimization of the synthesis protocol, beads produced from both 1.5% and 2% w/v alginate solutions were stable (did not collapse or rupture) up to 28 days. 2% alginate beads synthesized with 21 gauge needles were most favourable as they exhibited smaller diameters and less swelling.

### 3.2. Survival and protection of SH-SY5Y cells in alginate hydrogels

Based on previous work, the addition of a polycation coating, an extra polymeric layer or a combination of both can lower the surface roughness compared to conventional cell-loaded alginate beads.^35^ By using a polycation coating, followed by the addition of an external layer, beads allowing diffusion within a specific molecular cut-off can be produced, the hypothesis being that the material will exclude key components of the immune system from reaching the encapsulated cells without restricting the diffusion of nutrients and release of bioactive agents.

**Fig. 4:**
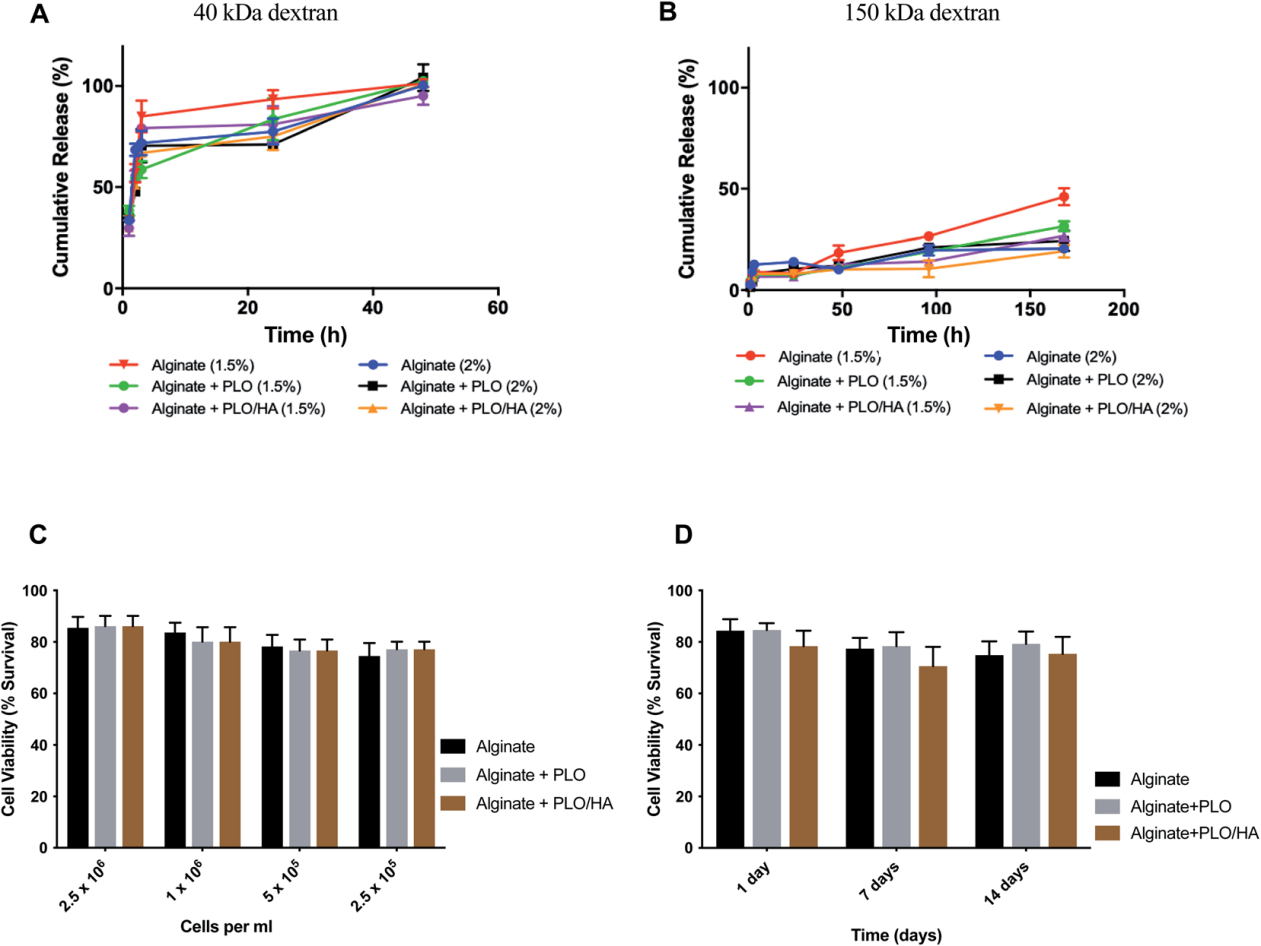
Alginate beads allow the diffusion of nutrients and can encapsulate neuronal cells without compromising cell viability. (A) Release of 40 kDa dextran from alginate beads alone, PLO coated, and PLO/HA coated for both 1.5% and 2% alginate. (B) Release of 150 kDa dextran from alginate beads alone, PLO coated, and PLO/HA coated for both 1.5% and 2% alginate. Data expressed as means ± SD, *N* = 3. (C) SH-SY5Y viability (2.5 × 10^5^ cells per ml to 2.5 × 10^6^ cells per ml) after 24 h in 2% alginate beads alone, with PLO or with PLO/HA. Two-way ANOVA followed by Bonferroni's multiple comparison test non-significant for different materials and significant for different cell densities (*p* < 0.0001). (D) SH-SY5Y viability up to 14 days in alginate beads alone, with PLO or with PLO/HA. Two-way ANOVA followed by Bonferroni's multiple comparison test significant for both different materials (alginate, alginate + PLO, and alginate + PLO/HA; *p* < 0.01) and time points (1, 7 and 14 days; *p* < 0.001).

Solute and antibody permeability was modelled by measuring the release of 40 kDa and 150 kDa molecular weight dextran from beads made from 1.5% and 2% w/v alginate solutions with and without additional surface coatings (PLO or PLO/HA). These studies demonstrated that the rate of molecular diffusion in the beads is a function of both the molecular weight of diffusing molecules as well as the initial alginate concentration and the presence of additional coating. In agreement with previous reports, the rates of diffusion increase as the initial concentration of the polymer decreases.^58,59^ In the case of 40 kDa dextran, the molecule diffused through the matrix easily, with the whole amount released after 48 h of incubation for all conditions (Fig. 4A). In contrast, although there was a certain level of transport through the polymeric network for the high molecular weight 150 kDa dextran, its diffusion was much more restricted. Coating of the beads was found to reduce the release of 150 kDa dextran by providing an additional barrier. The extent of the effect varied between time points, with the highest difference observed between at 96 h and 168 h for 1.5% alginate (*p* < 0.0001 for alginate *vs.* alginate + PLO/HA) and at 96 h 2% alginate (*p* < 0.001 for alginate *vs.* alginate + PLO/HA) (Fig. 4B).

This suggests that beads are suitable for encapsulation of cells as they allow the transport of essential nutrients whilst protecting against the infiltration of immune cells. This is confirmed by the findings that both alginate compositions tested here supported SH-SY5Y cell survival (Fig. 4C and D). To test viability, cells were encapsulated in 2% alginate beads produced using a 21G needle and syringe pump for reproducibility. The increase of cell concentration from 2.5 × 10^5^ to 2.5 × 10^6^ cells per ml of alginate did not have a noticeable influence on cell survival (Fig. 4C). Moreover, encapsulation of SH-SY5Y cells sustained their viability at over 77% for up to 14 days (Fig. 4D). Our results are in accordance with the conclusions of previous work on the proliferation of neuronal cells embedded in soft alginate substrates.^60–62^ While the results are promising, future studies should investigate the long-term viability of cells in these materials *in vivo*.

### 3.3. Refining the microbead design to reduce the local immune response

**Fig. 5:**
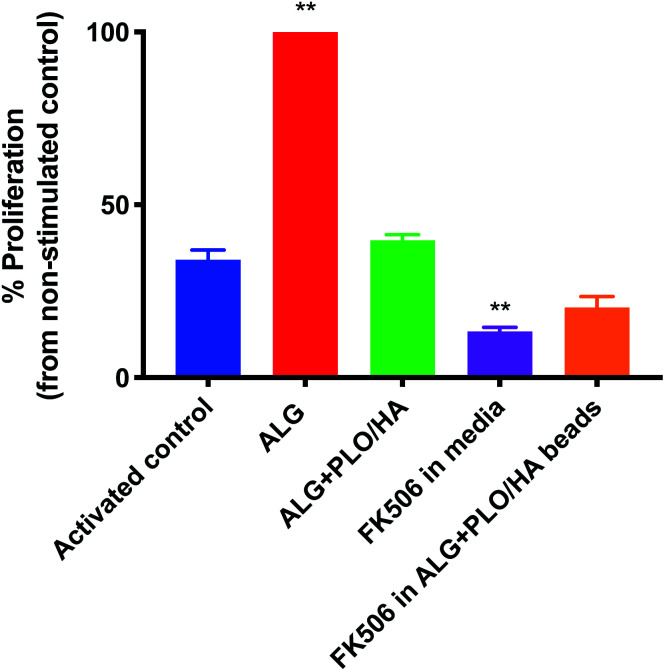
Ability of FK506 to reduce the potential immunogenic effect of alginate beads. Activated T-cells were exposed to 2% alginate beads alone, PLO/HA coated alginate beads, 100 ng ml^−1^ FK506 in the media or soluble FK506 encapsulated in alginate beads. After 4 days, proliferation was analysed by flow cytometry and the percentage of proliferation compared to the non-stimulated control was quantified. Data expressed as means ± SD, *N* = 3, with 3 repeats per condition, one-way ANOVA with Dunnett's multiple comparison test where **p < 0.01, all conditions compared to the activated control.

In addition to oxygen and nutrient supply and physical protection during injection, a key consideration for biomaterial encapsulation of implanted cells is positive modulation of the host tissue immune response. The extent to which alginate beads, modified or unmodified, were able to elicit an immune response was examined *in vitro* using a T-cell proliferation assay (Fig. 5). To our knowledge, the effect of alginate on T-cell proliferation has not been extensively studied. Unmodified alginate beads elicited a strong response compared to the control (65.9% increase). PLO/HA coating reduced the proliferation to 41.2 ± 1.7%. It should be noted that this proliferation index was comparable to the stimulated control, which exhibited 34.6 ± 2.9% cellular division, implying that alginate + PLO/HA beads are not strongly immunogenic.

To further reduce the immune response, we explored the co-administration of the immunomodulatory agent FK506, an approved immunosuppressive agent used for solid organ transplantation in humans. Neuroprotective and neurotrophic effects of FK506 have been previously demonstrated on dopaminergic neurons. For example, Castilho *et al.* have illustrated that treatment of rat embryonic dopaminergic neurons with immunophilin ligands FK506 and cyclosporin A is protective both *in vitro* and following grafting to the rat brain.^63^ Given its widespread use and proven efficacy in rejection prophylaxis for transplantation, FK506 is an appropriate drug candidate for incorporation into the beads. Furthermore, while FK506 has been used in the clinic for years, it has a narrow therapeutic index, variable bioavailability, and is associated with adverse effects and interactions.^64,65^ Therefore, local administration may be beneficial in terms of patient safety and increased efficiency. Fig. 5 shows that T-cell proliferation was further reduced by the addition of FK506 in free form and encapsulated in the bead coating (to 13.6 ± 1.2% and 21.2 ± 3.1% respectively), which implies that encapsulation of FK506 did not alter the ability of the drug to suppress the T-cell response.

**Fig. 6:**
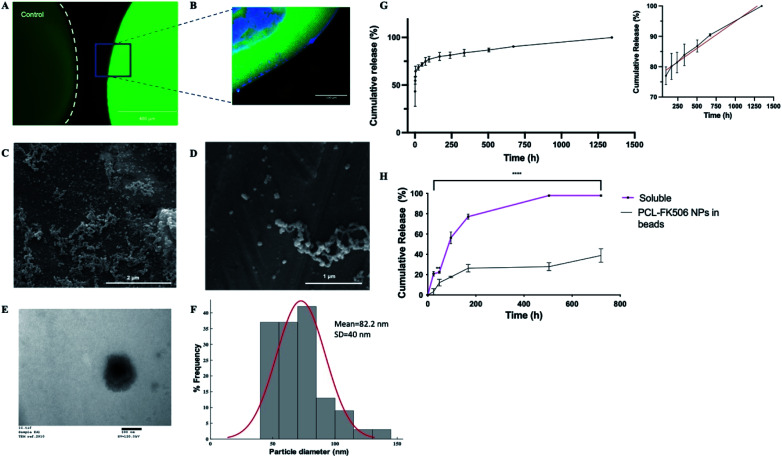
Controlled release of FK506 from implantable delivery system. (A) Fluorescence micrographs of control alginate bead and alginate bead with FITC positive PCL nanoparticles encapsulated in the HA-ALG coating, scale bar = 400 μm. (B) Confocal micrograph of section through the edge of an alginate bead with DAPI in the core and FITC positive PCL nanoparticles in the coating, scale bar = 100 μm. (C and D) Representative SEM images of PCL NPs, scale bars = 2 μm and 1 μm respectively. (E) Representative TEM image of PCL NPs, scale bar = 100 nm. (F) Histogram showing the size range of the nanoparticles. (G) Total cumulative release of FK506 from PCL nanoparticles over 2 months. The inset represents the linear release profile of FK506-nanoparticles (black line) and best fit zero-order release kinetics (red line) from 96 to 1344 h. Data expressed as mean ± SD (*N* = 3). (H) Release from alginate beads of FK506 incorporated either in solution or in nanoparticles within the outer layer. Data are expressed as mean ± SD (*N* = 3) with 3 repeats per condition, two-way ANOVA with Bonferroni's multiple comparison test where **p < 0.01 and ****p < 0.0001.

To provide local drug release, FK506 was incorporated in the outer layer of ALG + PLO/HA beads in soluble form or in nanoparticles (Fig. 6A and B). To prolong FK506 release from the beads, a novel drug delivery system was developed by loading the drug into core–shell nanoparticles (PCL-FK506 NPs) synthesized by coaxial electrospraying (Fig. 6C–F). Nanoparticles were successfully generated within a clinically acceptable size range (20–100 nm); not big enough to be destructive or small enough to be cleared.^66–69^ SEM and TEM images of the resulting nanoparticles can be seen in Fig. 6C–E. Mean size of the PCL-FK506 NPs was found to be 82 ± 40 nm (Fig. 6F). The entrapment efficiency of FK506 in PCL NPs was found to be 61.8%.

The drug release profile for the nanoparticles followed biphasic kinetics with approximately 60% of the drug being released during the initial burst (Fig. 6G). This could be attributed to the high surface area of the NPs. After the initial burst, a linear release profile was observed from 96 hours to 1344 hours (Fig. 6G inset). Electrosprayed PCL-FK506 nanoparticles were incorporated into the outer layer of the alginate beads, leading to a more sustained release compared with incorporation of FK506 in solution, with approximately 39% of the drug being released within 30 days (Fig. 6H). For FK506 incorporated in the outer layer of the beads in solution the release occurred in two phases, an initial slow-rate over the first 72 hours followed by a burst of release on the fourth day, and 97.5% of the drug was released by 336 hours (14 days). The longer-term prolonged release profile achieved by incorporation of FK506-loaded nanoparticles in the outer layer of the beads is particularly important as clinical trials of transplantation to the brain have shown that immunosuppression is required to prevent rejection and suggest that there is an optimum duration of immunosuppression which is required throughout the transplantation and observational period (typically at least 6 months); withdrawal of treatment too early affects functional outcomes.^70–72^

## 4. Conclusion

In summary, we have developed a novel alginate-based cell-encapsulation and drug delivery system that can maintain cell viability for up to 14 days *in vitro* as demonstrated with the SH-SY5Y cell line, with the potential to allow release of bioactive molecules while protecting implanted cells. Synthesis methods and material properties have been optimized for delivery to the brain, and a polymer coating incorporating nanoparticles for slow release of the immunosuppressant FK506 allows for reduction of the host immune response to implanted material and cells.

## Author contributions

DE and REE were responsible for the conceptualization, methodology and investigation and for drafting the manuscript. EA, AA and FG were responsible for data/evidence collection and formal analysis. ASB contributed to conceptualization and formal analysis. GRW and JCK contributed to methodology, writing – review and editing. VHR contributed to writing – review and editing and methodology. JBP provided supervision and guidance throughout.

## Conflicts of interest

None of the authors have any relevant conflicts of interest to declare.

## Supplementary Material
